# Circadian Rhythm of Blood Pressure of Dipper and Non-dipper Patients With Essential Hypertension: A Mathematical Modeling Approach

**DOI:** 10.3389/fphys.2020.536146

**Published:** 2021-01-18

**Authors:** Javiera Cortés-Ríos, Maria Rodriguez-Fernandez

**Affiliations:** Institute for Biological and Medical Engineering, Schools of Engineering, Medicine and Biological Sciences, Pontificia Universidad Catolica de Chile, Santiago, Chile

**Keywords:** mathematical model, blood pressure, circadian rhythm, dipper, non-dipper

## Abstract

Blood pressure in humans presents a circadian variation profile with a morning increase, a small postprandial valley, and a deeper descent during night-time rest. Under certain conditions, the nocturnal decline in blood pressure can be reduced or even reversed (non-dipper), which is related to a significantly worse prognosis than a normal fall pattern (dipper). Despite several advances in recent years, our understanding of blood pressure's temporal structure, its sources and mechanisms is far from complete. In this work, we developed an ordinary differential equation-based mathematical model capable of capturing the circadian rhythm of blood pressure in dipper and non-dipper patients with arterial hypertension. The model was calibrated by means of global optimization, using 24-h data of systolic and diastolic blood pressure, physical activity, heart rate, blood glucose and norepinephrine, obtained from the literature. After fitting the model, the mean of the normalized error for each data point was <0.2%, and confidence intervals indicate that all parameters were identifiable. Sensitivity analysis allowed identifying the most relevant parameters and therefore inferring the most important blood pressure regulatory mechanisms involved in the non-dipper status, namely, increase in sympathetic over parasympathetic nervous tone, lower influence of physical activity on heart rate and greater influence of physical activity and glucose on the systemic vascular resistance. In summary, this model allows explaining the circadian rhythm of blood pressure and deepening the understanding of the underlying mechanisms and interactions integrating the results of previous works.

## Introduction

Homeostatic regulatory mechanisms allow keeping physiological variables in the human body within acceptable ranges. These mechanisms operate all the time, generating dynamic variation profiles such as the blood pressure profile. Blood pressure is regulated mainly by the neurohumoral system that includes the renin-angiotensin-aldosterone system, natriuretic peptides of the endothelium, the sympathetic nervous system and the immune system. These regulatory mechanisms allow the human body to respond to changes in factors such as physical activity and diet (Peixoto and White, 2007). However, under physiopathological conditions, blood pressure can be continuously elevated, causing damage to the vessels and organs that it irrigates; this condition is known as arterial hypertension. Depending on the cause of this pathology, it can be classified as either primary or essential hypertension, which has no identifiable cause, or secondary hypertension, when it is possible to identify the pathology or condition that triggers the disease. The development of essential hypertension has been associated with family history and genetic predisposition and a set of factors related to lifestyle, such as mental stress, high salt intake, poor sleep quality or sleep apnea, and alcohol consumption, among others (Oparil et al., 2018).

Humans have an internal clock of ~24 h, which allows us to anticipate and prepare for events or disturbances that occur during the activity-rest cycle. Consequently, periodic changes in diet, sleep-wakefulness, behavioral cycles, and other physiological activities and processes occur daily in humans and other organisms (Feng and Lazar, 2012). Among the physiological variables that present a circadian variation, blood pressure is one of the most studied due to its impact on chronic cardiovascular diseases and complex events such as strokes and heart failure. This circadian variation profile is mainly related to the activity-rest cycle and presents a morning increase, a small postprandial valley, and a deeper descent during night-time rest. Under certain physiological conditions, the nocturnal decline in blood pressure can be reduced or even reversed (non-dipper pattern), which is related to a significantly worse prognosis than a normal fall pattern (dipper). Different hypotheses have been proposed about the causes of the development of a non-dipper pattern, including a renal mechanism due to high salt intake (Fukuda and Kimura, 2012), the alteration of the sleep-wake cycle (Kitamura et al., 2002), higher activity during night rest (Mansoor, 2002) and the relationship of the non-dipper pattern with a decrease in the parasympathetic nervous function and an increase in the sympathetic nervous function (Nakano et al., 2001). However, the causes of the development of the non-dipper pattern are still unclear. The integration of knowledge about the physiological mechanisms of control and the variables that alter blood pressure through mathematical models would allow us to further understand the dynamics of the system to improve the diagnosis and treatment of this disease.

Several methods have been proposed for the characterization of the circadian profile of blood pressure, ranging from simple statistics including the median and mean values (Parati, 2004) to more complex techniques such as Fourier analysis (Staessen et al., 1993), cosinor mixed-model analyses of variance (Shea et al., 2011), modeling of non-linear mixed effects (van Rijn-Bikker et al., 2013) and multiple-components analysis (Hermida et al., 2002a). However, the aforementioned models allow modeling the circadian rhythm of blood pressure based on data rather than physiological regulatory mechanisms. Therefore, they do not allow deepening into the mechanisms involved in the blood pressure drop during the night-time rest. The first mathematical model that sought to explain blood pressure variation through physiological control mechanisms was developed by Guyton et al. (1972). The model consists of hundreds of mathematical equations designed primarily to understand the long-term regulation of blood pressure and cardiac output and has served as inspiration and basis for many other integrative models of physiology. Other models were developed to explain the short-term response of the circulatory system; for example, the orthostatic response to head-up tilt (Melchior et al., 1992; van Heusden et al., 2006). More recently, Albanese et al. (2016) developed an integrated mathematical model of the human cardiopulmonary system. Other authors developed a mathematical model of salt-sensitive hypertension that challenges Guyton model's assumptions and does not limit the cause of salt-sensitive hypertension to primary renal dysfunction only since it includes the possibility of being the result of a neurogenic dysfunction (Averina et al., 2015). Despite the capabilities of the Guyton model and subsequently revised models to capture the dynamics of blood pressure at different time scales, they do not adequately characterize the circadian variation of blood pressure.

This work's objective was to develop a mathematical model using ordinary differential equations (ODEs) based on the mechanisms of physiological control of blood pressure to explain the circadian variation profile of dipper and non-dipper patients with essential hypertension. The development of this model allowed explaining dipper and non-dipper patterns integrating the results of previous works that attempted to explain differences between these subjects, evaluating different physiological variables.

## Methods

### Foundations of the Mathematical Model

The mathematical model was based on the expected physiological response resulting from the interaction between the different variables involved in the medium-term blood pressure regulation. Several mechanisms are known to be involved in the control of blood pressure. However, to simplify the model and ensure identifiability, fundamental inputs that have more significant influence or represent the behavior of a set of variables that affect the circadian variation in blood pressure were used in this work. The chosen input variables included in the model are norepinephrine (NE), physical activity (A), and glycemia (glc), and the dependent variables are systemic vascular resistance (SVR), systolic blood pressure (SBP), diastolic blood pressure (DBP), and heart rate (HR).

Physical activity was chosen since previous works have established that it is one of the determinants of the variation in blood pressure and the variability of heart rate (Kario et al., 1999; Leary et al., 2000; Mansoor, 2002). Furthermore, greater night-time activity has been observed in non-dipper patients, making it one of the influencing factors in altering the circadian profile. However, it has been concluded that it cannot predict a non-dipper pattern by itself (Hermida et al., 2002b). Therefore, other variables related to the alteration of the circadian pattern of blood pressure were also considered. On the one hand, the plasma levels of norepinephrine exhibit a circadian variation, increasing during the day and decreasing during night time rest (Linsell et al., 1985; Candito et al., 1992). It is known that norepinephrine generates vasoconstriction and increases the cardiac frequency and output due to its action on adrenergic receptors. Furthermore, a high correlation has been shown between plasma norepinephrine and the outflow of the sympathetic nervous system (Goldstein et al., 1983), which is part of the most relevant blood pressure regulation mechanisms. For example, renin release has been shown to be influenced by sympathetic outflow (Zanchetti and Stella, 1975) and a direct relationship between norepinephrine levels and plasma renin activity has been observed in both healthy and hypertensive subjects (Beretta-Piccoli et al., 1980; Tuck et al., 1985). Therefore, by including this variable, we include its direct effect and, indirectly, other relevant regulatory mechanisms. Moreover, the variations of this catecholamine have been related to the circadian variation profile of blood pressure and a positive correlation has been observed between urine norepinephrine and the fall in blood pressure at night rest (Arita et al., 1996; Därr et al., 2016). On the other hand, it has been shown that a sharp increase in plasma glucose can decrease peripheral vascular resistance by 13%, increase heart rate by 14%, consequently increase 20% of cardiac output, maintaining unaffected blood pressure (Jern, 1991). Furthermore, blood glucose and the consequent increase in insulin have a positive effect on the sympathetic nervous system without altering plasma norepinephrine (Rowe et al., 1981). Additionally, differences in glucose profiles have been observed between dipper and non-dipper patients with type II diabetes, where non-dipper patients have been shown to have greater insulin resistance (Pistrosch et al., 2007). Differences have also been observed between dipper and non-dipper patients with essential hypertension, where those with a non-dipper pattern show greater insulin resistance (Chen et al., 1998).

### Mathematical Model

**Figure 1:**
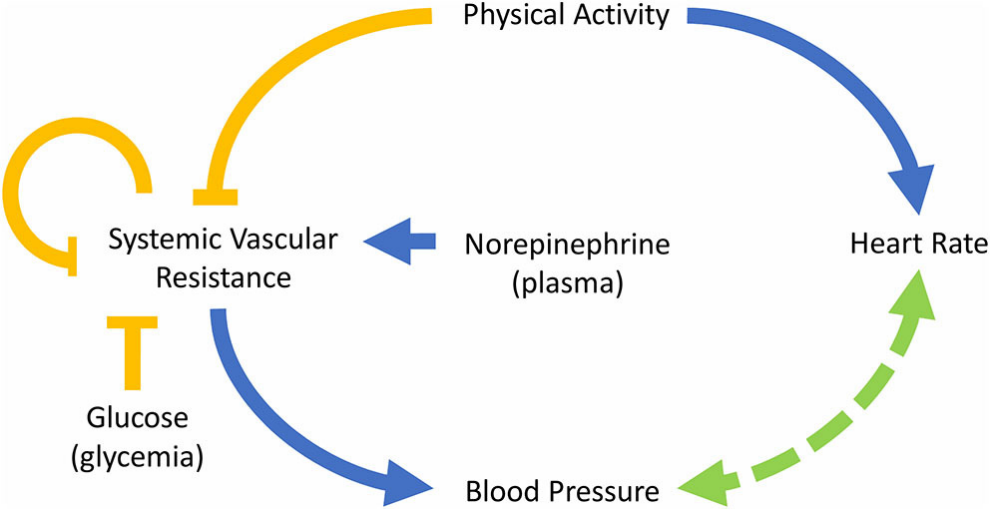
Diagram of the model describing the interactions between the main variables involved in the control of blood pressure. The blue arrows indicate “activation,” the yellow arrows “inhibition” and the arrow with segmented line refers to a mutual regulation.

The equations were formulated based on the dynamics of the interactions described in the scheme of Figure 1. Direct relationships were modeled by means of first-order kinetics while higher-order interactions involving activating or inhibiting effects were modeled using Hill functions. For the sake of parsimony, a Hill coefficient equal to one was considered when possible. The effects described between variables do not necessarily imply a direct mechanism of activation or inhibition but express the expected physiological response that each of the involved variables exerts on one another. For example, the relationships between physical activity and heart rate and between physical activity and SVR are complex and include several components and regulation systems; however, we have simplified these relationships considering only the initial and final expected effects. Specifically, physical activity generates a greater demand for oxygen and nutrients in the skeletal muscle; this causes the muscle to release vasodilators such as nitric oxide, which reduces vascular resistance and blood pressure in that area. Then, the sympathetic nervous system acts to regulate blood pressure by increasing the vascular resistance in the abdominal viscera and increasing heart rate and stroke volume, thereby increasing cardiac output and, consequently, blood pressure (Nobrega et al., 2014; White and Raven, 2014). Despite the increase in vascular resistance in the visceral abdominal area, its decrease at the peripheral level generates a general reduction in vascular resistance (Casey and Joyner, 2012). The relationships described above are reflected in the SVR and HR equations (Equations 1, 3) by the expressions $\frac{k_{i1}}{k_{i1}+A}$ and $\frac{A}{k_{i4}+A}$, which represent the inhibition and activation exerted by physical activity on the SVR and HR variables, respectively. Therefore, the parameters *k*_*i*1_ and *k*_*i*4_ are constants that indicate the relevance of the effect of the activity in the changes of SVR and HR, respectively. The larger the value of *k*_*i*1_, the smaller the regulation (inhibition) of the SVR due to the activity. Analogously, the larger the value of *k*_*i*4_, the smaller the regulation (activation) of the HR due to the activity.

Several mechanisms regulate vascular resistance. Some regulatory molecules are capable of generating a short-term response; for example, the action of catecholamines, like norepinephrine, on alpha-adrenergic receptors causes vasoconstriction (Takeda and Maemura, 2011; Tank and Lee Wong, 2015). Moreover, as mentioned above, norepinephrine has a high correlation with the outflow of the sympathetic nervous system (Goldstein et al., 1983), which is related to vasoconstriction. On the other hand, insulin secretion, triggered by increased blood glucose, generates vasodilation (Muniyappa et al., 2007). Hence, the influence of both norepinephrine and glucose was included in the model on the expression $\frac{NE}{glc^{n}+NE}$. In a previous version of the model, the effect of glucose and norepinephrine was modeled by additive inhibition and activation expressions, respectively. However, a better fit to the data was obtained by relating them in a single expression, suggesting a synergistic effect. Therefore, the parameter *n* is a constant that represents the impact of glucose on vasodilatation and *k*_1_ represents the basal rate of vasoconstriction (without the influence of the activity and the glucose). Finally, the expression $\frac{SVR}{k_{i2}+SVR}*SVR$, that represents the regulation mechanism of vascular resistance of blood vessels produced when blood flow to an organ decreases generating metabolic-induced vasodilatation (Nobrega et al., 2014), was also included in Equation (1). Consequently, the *k*_*i*2_ value is related to the self-regulatory capacity of SVR.

Equations (2) and (4) describe the change of blood pressure over time, which depends directly on SVR because, for a given blood flow, an increase of vascular resistance generates an increase in the pressure on the blood vessels' walls (SBP and DBP). In addition, the influence of BP over HR is captured by the expression $\frac{HR}{k_{i3}+HR}$; a higher HR (together with a higher stroke volume) generates an increase in cardiac output, increasing blood flow, which also induces an increase in SBP and DBP. Due to this, the parameter *k*_*i*3_ is related to the ability of HR to modify the value of BP. Besides, heart rate is regulated by the autonomic nervous system, which activity at this level is regulated, in part, by the activation of baroreceptors, that are able to detect changes in BP (Guyenet, 2006). This relationship between BP and HR is known as baroreflex regulation, and it was included in Equations (2)–(4). Baroreflex regulation allows regulating the values of HR and BP, increasing the value of HR when BP decreases and vice versa. Finally, a constant rate of increase of HR or baseline is expressed by *k*_2_. Moreover, parameters k_3_ and k_4_ were incorporated into the model to ensure dimensional homogeneity.

Thus, the following system of four differential equations and seven parameters was obtained:

(1) $$\begin{array}{l} \frac{dSVR}{dt}=k_{1}*\frac{k_{i1}}{k_{i1}+A}*\frac{NE}{glc^{n}+NE}-\frac{SVR}{k_{i2}+SVR}*SVR \end{array}$$

(2) $$\begin{array}{l} \frac{dSBP}{dt}=k_{3}*SVR*\frac{HR}{k_{i3}+HR}-SBP \end{array}$$

(3) $$\begin{array}{l} \frac{dHR}{dt}=k_{2}*\frac{A}{k_{i4}+A}-k_{4}*DBP*HR \end{array}$$

(4) $$\begin{array}{l} \frac{dDBP}{dt}=k_{3}*SVR*\frac{HR}{k_{i3}+HR}-SBP \end{array}$$

Some variables, as changes in fluid volume, were not considered due to unavailability of data; however, these variables were enough to capture the general pattern of the circadian rhythm of blood pressure.

### Experimental Data

Experimental data on physical activity, heart rate, and blood pressure (systolic and diastolic) for dipper and non-dipper subjects measured every 30 min over 24-h were extracted using the Engauge Digitizer software from the work of Hermida et al. (2002b). The data correspond to the average of 130 male patients (53.7 ± 14.0 years of age).

The norepinephrine data for dipper patients was extracted from the work of Linsell et al. (1985) and van Dijk and van Loon (2015) and the values for non-dipper patients were simulated by increasing by 15% during the night rest the values obtained from dipper patients, according to the work of Därr et al. (2016), who reported a significant difference between day-night urinary norepinephrine excretion ratio between dippers and non-dippers.

The glycemia data for dipper patients was extracted from the work of van Dijk and for non-dipper patients, these values were increased by 4.7, 13.3, and 8.1% 2 h after each of the three meals: breakfast, lunch, and dinner, respectively, according to what was reported by Pistrosch et al. (2007).

The systemic vascular resistance (SVR) data to fit the model was obtained using the following equation (Coats et al., 1989):

(5) $$\begin{array}{l} SVR_{\left(dyn*\frac{s}{cm^{5}}\right)}\;=\frac{\left(\frac{2*SBP+DBP}{3}-CVP\right)*80}{SV*HR} \end{array}$$

assuming an average stroke volume (*SV*) for subjects with essential hypertension of 79.5 ml (Messerli et al., 1983) and replacing the systolic blood pressure (SBP), diastolic blood pressure (DBP) and heart rate (HR) of dipper and non-dipper subjects from the work of Hermida et al. (2002b). The value of the mean right atrial pressure for essential hypertension patients (5 mmHg) (Ferlinz, 1980) was used instead of the central venous pressure (CVP), since, as previously reported, both values are similar given the low resistance of large vessels (Magder, 2005). The standard deviation of SVR was calculated from the standard deviation of the variables SBP, DBP and HR.

The data were organized in such a way that the simulation begins at 0 a.m. and wake-up time is 8 a.m. for all the data.

### Parameter Estimation

The ODE-based model was developed using previous knowledge about the main regulatory mechanisms of blood pressure, detailed in the Mathematical Model and Experimental Data sections, and was implemented in MATLAB 2018a. Each model's parameters were fitted to the experimental data employing global optimization using a scatter search method (MEIGO) (Egea et al., 2014). Scatter Search is an evolutionary meta-heuristic able to solve combinatorial and non-linear optimization problems, which allows fitting the model to the experimental data in order to obtain the values of the parameters that minimize an objective function, i.e., given a certain range for each parameter, MEIGO iteratively searches for the combination of the parameters that minimize the error between the experimental data and the model predictions. To integrate the model, the solver ode23s of MATLAB was chosen and the objective function is expressed as a sum of squares:

(6) $$\begin{array}{l} F_{objetive}=\frac{1}{k*N}\overset{k}{\underset{i=1}{\sum }}\overset{N}{\underset{n=1}{\sum }}{\left(\frac{y_{\exp i}\left(t_{n}\right)-y_{\text{model }i}\left(t_{n}\right)}{y_{\exp i}\left(t_{n}\right)}\right)}^{2} \end{array}$$

where N is the number of experimental data per variable, *k* is the number of variables, and *y*_exp *i*_ is the experimental value of the *i* variable, and *y*_model *i*_. is the value predicted by the model. Twenty-four hour data were used to adjust the model.

Data from both types of subjects were used independently to estimate the parameters. Then, according to the values obtained individually, some parameters were established in common for both types of subjects and the remaining parameters were re-fitted.

### Residual Analysis

The analysis of residuals was carried out using Equation (7), where the residuals, *R*(*t*_*n*_), are calculated using the experimental values (HR, SBP and DBP), *y*_expNC*i*_(*t*_*n*_), and the values predicted by the model, *y*_model*i*_(*t*_*n*_), at each time, *t*_*n*_.

(7) $$\begin{array}{l} R\left(t_{n}\right)=y_{\text{expNC}i}\left(t_{n}\right)-y_{\text{model }i}\left(t_{n}\right) \end{array}$$

To evaluate the residuals, a QQ plot and a histogram were plotted using MATLAB's *qqplot* and *hist* functions. Furthermore, to corroborate these results, the Shapiro Wilks test of normality was used using MATLAB's *swtest* function with an alpha value of 0.05.

### Sensitivity Analysis

Local sensitivity analysis allows quantifying the impact of the local variation of a parameter on the value of each of the output variables (in contrast with global sensitivities that explore the entire parameter space). In this work, the local sensitivity of each variable *y*_*i*_ with respect to each parameter *p*_*j*_ evaluated for the best parameter set was computed using the SENS_SYS third-party MATLAB function (Garcia Molla, 2019):

(8) $$\begin{array}{l} S_{ij}\left(t_{n}\right)=\frac{dy_{model\;i}\left(t_{n}\right)}{dp_{j}} \end{array}$$

Then, normalization was performed using the following equation:

(9) $$\begin{array}{l} Srel_{ij}\left(t_{n}\right)=\frac{dy_{model\;i}\left(t_{n}\right)}{dp_{j}}*\frac{p_{j}}{y_{model\;i}\left(t_{n}\right)} \end{array}$$

*Srel*_*ij*_(*t*_*n*_) was calculated for each parameter (j), variable (i), and time point (*t*_*n*_) obtaining relative sensitivity trajectories for each parameter and each variable (SVR, SBP, HR, and DBP). The analysis of these results is complex, due to the large number of sensitivity values (for each time and variable). Therefore, a sensitivity index for each parameter was calculated using the following equation (Brun et al., 2001), which transforms the relative sensitivity trajectories corresponding to each parameter into positive values by squaring and averaging them across the different time points and variables:

(10) $$\begin{array}{l} \delta_{j}=\sqrt{\frac{1}{k*N}\overset{k}{\underset{i=1}{\sum }}\overset{N}{\underset{n=1}{\sum }}Srel_{ij}^{2}\left(t_{n}\right)} \end{array}$$

where N is the number of experimental data per variable, *k* is the number of variables, and *y*_model *in*_ is the value *n* of the *i* variable predicted by the model.

### Identifiability Analysis

To carry out an identifiability analysis, the Fisher information matrix (FIM) was obtained from the local sensitivity matrix *S*(*t*_*n*_) and the covariance matrix *Q*(*t*_*n*_) (Ljung, 1999):

(11) $$\begin{array}{l} FIM=\overset{N}{\underset{n=1}{\sum }}S\left(t_{n}\right)\cdot Q\left(t_{n}\right)\cdot S{\left(t_{n}\right)}^{T} \end{array}$$

where *Q*(*t*_*n*_) is a 4-by-4 diagonal matrix calculated using the standard deviation of the data for SBP, DBP, HR, and the estimated standard deviation for SVR at each time point and *S*(*t*_*n*_) is a 7-by-4 matrix obtained from the local sensitivity values of each of the four variables with respect to each of the seven parameters at each time point (Equation 8).

According to the Cramér–Rao theorem, the inverse of the FIM represents an approximation of the covariance matrix of the error of the objective estimator of minimum variance (Ljung, 1999). In this case, the inverse of the FIM is a 7-by-7 matrix where each element represents an approximation of the parameter estimation error covariance between the parameters j and h ($\sigma_{jh}^{2}$):

(12) $$\begin{array}{l} \sigma_{jh}^{2}={\left(FIM^{-1}\right)}_{jh} \end{array}$$

Therefore, the diagonal of the inverse of the FIM $\left(\sigma_{jj}^{2}\right)$ is an approximation of the variance of the parameters. Thus, assuming a normal distribution, the 95% confidence intervals (CI) of a parameter can be approximated by *p* ± 1.96 σ_*jj*_ (Walter and Pronzato, 1997).

Moreover, the correlation between parameters (κ_*jh*_) can be calculated using the following equation (Ljung, 1999):

(13) $$\begin{array}{l} \kappa_{jh}=\frac{\sigma_{jh}^{2}}{\sigma_{hh}\cdot \sigma_{jj}} \end{array}$$

The correlation matrix measures the interrelationship between the parameters and gives an idea of the compensation effects of changes in the parameter values on the output variables. If two parameters are highly correlated, a change in the model output caused by a change in a model parameter can be (nearly) compensated by an appropriate change in the other parameter value. This prevents the parameters from being uniquely identifiable even if the model output is very sensitive to changes in the individual parameters. A singular FIM indicates the presence of unidentifiable parameters, and correlations between parameters that are >0.95 may lead to singular FIM.

## Results

### Parameter Estimation

**Figure 2:**
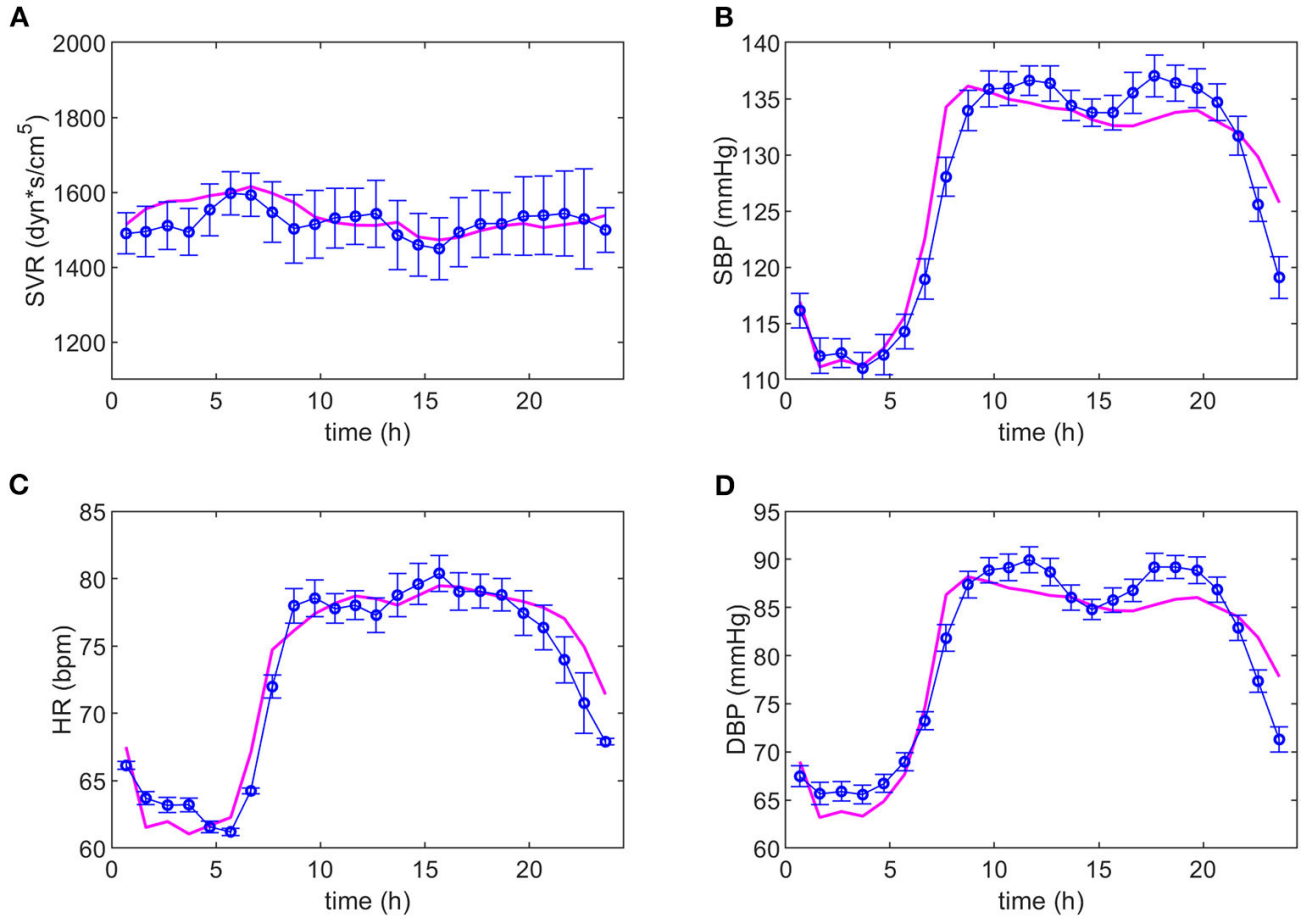
Adjustment of the model to the experimental data of dipper subjects. The magenta line represents the values predicted by the model and the blue line, the experimental data with their respective standard deviations. Dynamics of systemic vascular resistance (SVR), systolic blood pressure (SBP), heart rate (HR), and diastolic blood pressure (DBP) are presented in **(A–D)**, respectively.

**Figure 3:**
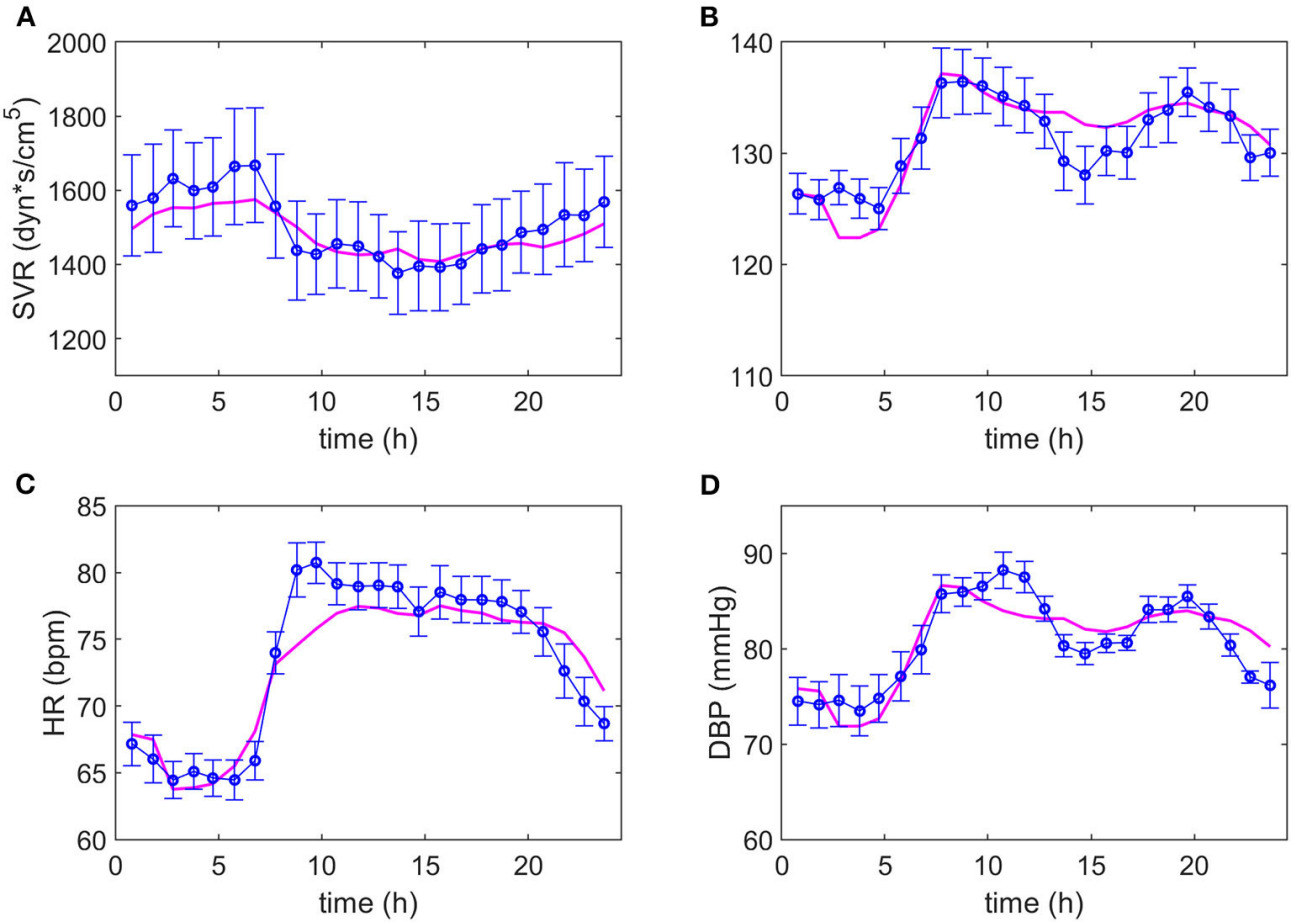
Adjustment of the model to the experimental data of non-dipper subjects. The magenta line represents the values predicted by the model and the blue line and markers, the experimental data with their respective standard deviations. Dynamics of systemic vascular resistance (SVR), systolic blood pressure (SBP), heart rate (HR) and diastolic blood pressure (DBP) are presented in **(A–D)**, respectively.

After several iterations in the search for equations representing the data, it was possible to adjust the same model to both dipper and non-dipper subjects. The experimental data and the model predictions for the dipper and non-dipper subjects can be seen in Figures 2, 3, respectively. The estimated values and 95% CI of the parameters are shown in Table 1. The parameters k_3_ and k_4_ were fixed to one due to its high correlation with k_i3_ and k_2_, respectively; they were kept in the model to ensure dimensional homogeneity. The table shows the common parameters between dipper and non-dipper subjects (k_1_, n, k_i2_, k_3_, and k_4_) and the parameters with different values for each type of subject (k_i1_, k_i3_, k_i4_, k_2_).

**Table 1:** Estimated parameters for dipper and non-dipper subjects.

**Parameter**	**Dipper value**	**CI 95%**	**Non-dipper value**	**CI 95%**
		**dipper (±)**		**non-dipper (±)**
k_3_	1	–	1	–
k_4_	1	–	1	–
k_1_	614.7	376.0	614.7	324.2
N	2.472	0.336	2.472	0.394
k_i2_	3,861	3,296	3,861	2,938
k_i1_	801.3	219.8	544.2	188.3
k_i3_	805.3	15.70	748.4	26.1
k_i4_	24.79	1.131	10.86	1.336
k_2_	7,654	105.8	6,808	122.3

The parameters k_1_, n, k_i1_ and k_i2_ are part of Equation (1), of which only k_i1_ presents a different value between both types of subjects, suggesting a difference in the SVR response to physical activity in non-dipper subjects. On the other hand, in Equation (2), a greater value for parameter k_i3_ was obtained for dipper subjects. This can be due to the range of HR values that is slightly smaller in non-dipper subjects (minimum value is lower in dipper subjects). In Equation (3), parameters k_i4_, k_2_ have higher values for dipper subjects, which can be explained by a higher activation baseline that requires a smaller increase in each time interval and a greater influence of physical activity on HR. The 95% confidence interval values indicate that all parameters are statistically significant. The value of the objective function was 0.0015; namely, the mean of the normalized error for each data point is 0.15%.

### Residual Analysis

The QQ plot and histogram of the residuals (Supplementary Figures 1, 2) show a distribution close to normal. This was verified using the Shapiro Wilks test, which indicates that, with a 95% confidence, the residuals come from a normal distribution (*p*-value = 0.762).

### Sensitivity Analysis

**Figure 4:**
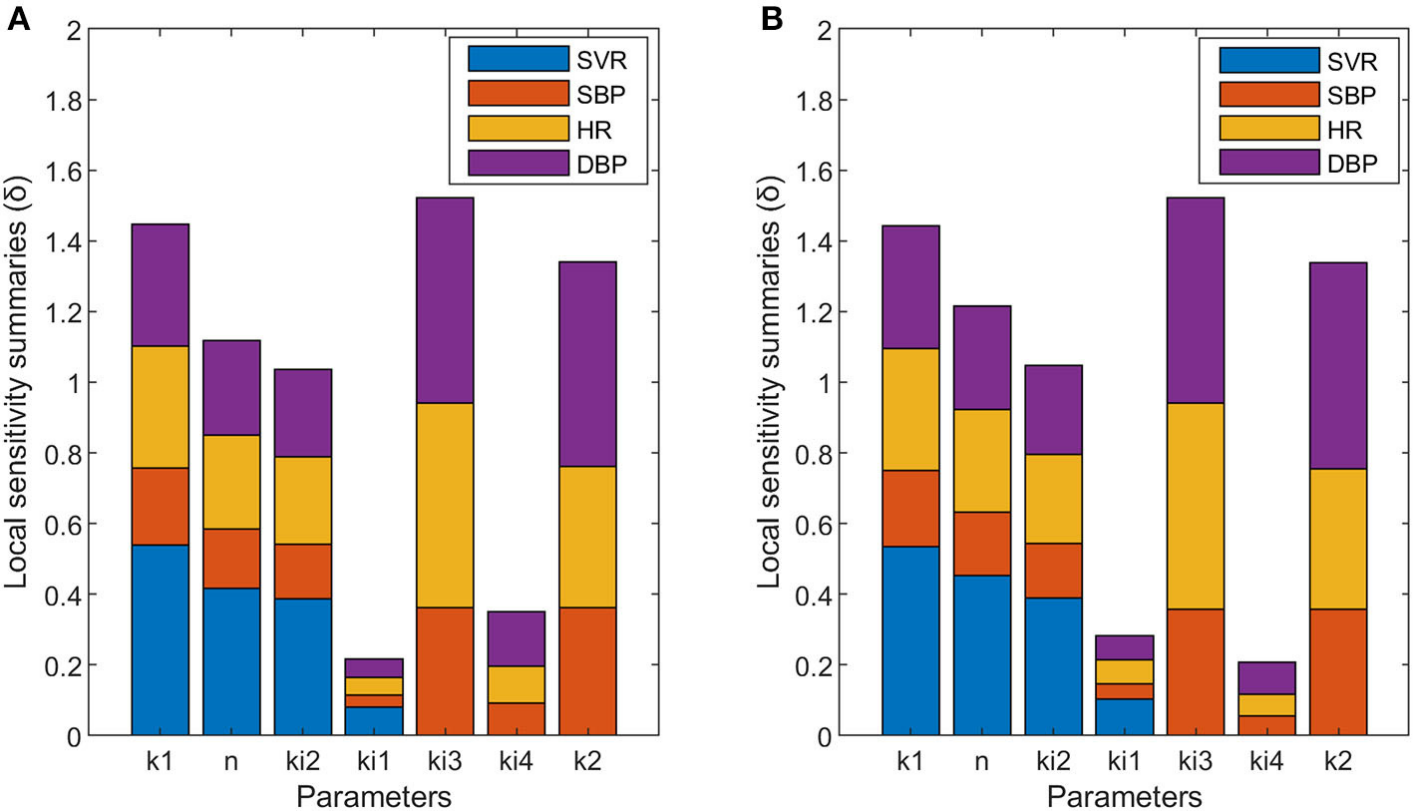
Local sensitivity summaries (δ) calculated for each parameter with respect to each variable designated with different colors: SVR (blue), SBP (orange), HR (yellow), and DBP (purple) of dipper **(A)** and non-dipper **(B)** subjects.

The results obtained for the sensitivity analysis are summarized in Figure 4. Parameters n and k_i1_ have higher sensitivity for non-dipper subjects than for dipper subjects, parameters k_1_, k_i2_, k_i3_, and k_2_ have similar sensitivity for dipper and non-dipper subjects, and parameter k_i4_ has higher sensitivity for dipper subjects than for non-dipper subjects. Parameters k_i1_ and k_i4_ have different sensitivity and they are related to physical activity, which indicates a different response to physical activity for non-dipper subjects. Additionally, the higher sensitivity of parameter n can be due to higher glycemia (glc) values for non-dipper subjects.

### Identifiability Analysis

**Figure 5:**
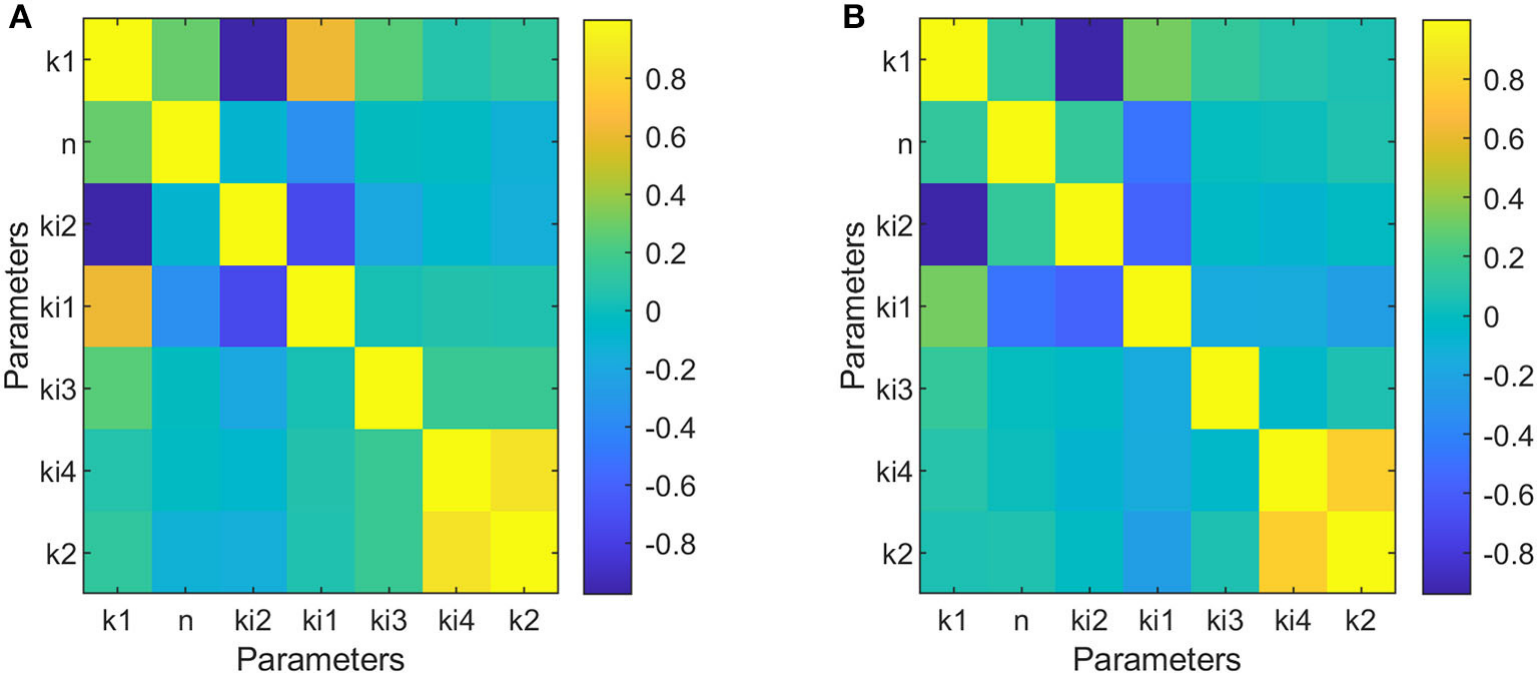
Correlation matrices of parameters adjusted to data of dipper subjects **(A)** and to data of non-dipper subjects **(B)**.

The results obtained for the identifiability analysis are summarized in Figure 5. The FIM matrix is not singular; therefore, it was possible to calculate its inverse and obtain an approximation of the correlation matrix for parameters of dipper subjects (Figure 5A) and non-dipper subjects (Figure 5B). Both types of subjects showed a high correlation between k_1_ and k_i2_, but only dippers subjects had a correlation value >0.95. The correlation between the rest of the parameters was <0.95. Therefore, the confidence intervals of the estimated parameters indicate that the model is capable of representing the data of dipper and non-dipper subjects with essential hypertension.

## Discussion

In this work, a model capable of capturing the blood pressure profile of dipper and non-dipper subjects is proposed. The results obtained show different values between the two groups for the parameters k_i1_, k_i3_, k_i4_, and k_2_, and different sensitivities for the parameters n, k_i1_, and k_i4_. In the case of parameters k_1_ and k_i2_, they present equal sensitivity and the same values for both dipper and non-dipper subjects. But, parameter n has equal value and greater sensitivity in non-dipper patients. This is related to the higher glycemic values and the narrower range of norepinephrine values of non-dipper subjects (Pistrosch et al., 2007; Därr et al., 2016), and explains the more abrupt drop in postprandial blood pressure in these subjects. Besides, the greater sensitivity of the parameters related to physical activity and glucose in the SVR equation in non-dipper subjects (k_i1_ and n) indicates a greater influence of physical activity and glucose on SVR; namely, as the glucose and physical activity increase, the SVR decreases more in non-dipper than in dipper subjects.

In the proposed model, predicted SVR fluctuations move within a wider range in non-dipper patients, presenting marked increases and decreases. This could be due to an altered function of vasoconstriction control, since the decrease in sympathetic control of vasoconstriction (sympathoinhibition) with high levels of sympathetic activity has been related to post-execution hypotension in patients with hypertension (Halliwill, 2001). Considering these precedents, the greater sensitivity of the parameter k_i1_ of non-dipper patients (which indicates greater sensitivity to physical activity) is not entirely surprising and indicates that, in non-dipper patients, there is an imbalance in the autonomic nervous system, i.e., an increase in the sympathetic over the parasympathetic tone in non-dipper subjects. This coincides with what was reported by Hojo et al. and Nakano et al., which suggested a decrease in the sympathovagal balance, with an acceleration of sympathetic function and a deceleration of parasympathetic function in non-dipper subjects with essential arterial hypertension (Hojo et al., 1997; Nakano et al., 2001).

On the other hand, when comparing SVR dynamics to HR dynamics, it can be noted that the SVR's behavior has an inverse tendency to that of HR, mainly in non-dipper patients, i.e., when HR increases, SVR decreases. This is related to the control of blood pressure through the baroreflex mechanism. In the model, the parameter k_i3_, which relates HR to BP in Equations (2) and (4), has equal sensitivity in both types of subjects but a greater value in the case of dipper subjects. This could indicate a greater influence of SVR than HR on the regulation of BP in non-dipper subjects. In addition, the value of k_2_ is lower in non-dipper subjects, which coincides with a greater sympathetic basal stimulation of heart, leading to higher HR values in non-dippers. Besides, the lower sensitivity of the k_i4_ parameter in non-dipper subjects indicates a lower influence of physical activity on heart rate in non-dippers. This coincides with the findings of Robinson et al. (1966), who studied the effect of the autonomic nervous system on HR and observed minor changes in HR when a parasympathetic block was performed at low levels of physical activity. The foregoing reinforces the idea of deregulation (at various levels of action) of the autonomic nervous system in non-dipper subjects, which has been reported previously (Grassi et al., 2010). In general, the greatest differences in the sensitivity of the parameters between both types of subjects are related to the influence of physical activity on the SVR and HR variables. Concerning the effect of physical activity on the variables studied, it has been reported that there is a decrease in norepinephrine levels and a reduction in sympathetic inhibition at the level of skeletal muscle after exercise training (Rengo et al., 2014; Besnier et al., 2017). In addition, Ling et al. (2015) reported a change in blood pressure dipper status in response to chronic exercise in African American non-dippers. Therefore, this background and the results of this work suggest that sedentary behavior patterns could be related to non-dipper subjects' profiles. The coincidence between our sensitivity analysis and parameter estimation results with previous research findings supports the proposed model and allows us to integrate these physiological findings into a mathematical model.

In summary, the mathematical model proposed here is able to reproduce the behavior of the circadian rhythm of blood pressure through ODEs using physical activity, glucose and norepinephrine as inputs. The objective of this model is not only to predict blood pressure, but to understand its regulation through the variation of physiological variables and thus understand the different profiles of blood pressure. However, the model presents several limitations that must be considered: (i) average data of different subjects were used, (ii) data from different works with sample sizes and different populations were used, (iii) many variables were not considered to avoid identifiability issues, and (iv) the parameters obtained do not necessarily imply causal associations and may reflect the interaction of the variables used with other unmeasured physiological variables. Despite these limitations, the adjusted model allowed predicting blood pressure with a mean of the normalized error of 0.15% for each data point. Finally, the development of this model allowed integrating the findings of several investigations that attempted to explain the different profiles of blood pressure from changes in individual physiological variables and the residual analysis using the non-calculated variables showed a normal distribution, which further supports the model assumptions.

## Data Availability Statement

Data generated during this study are available from the corresponding author upon reasonable request.

## Author Contributions

All authors listed have made a substantial, direct and intellectual contribution to the work, and approved it for publication.

## Conflict of Interest

The authors declare that the research was conducted in the absence of any commercial or financial relationships that could be construed as a potential conflict of interest.

## Figures and Tables

**Table:** 

F_objective_	Objective function
*Srel*_**ij**_(*t*_**n**_)	Relative local sensitivity of *x*_**i**_ variable to variations of parameter j at time t_n_
t_n_	Time of the experimental data number n
*k*_**jh**_	Correlation between j parameter and h parameter
*p*_**j**_	Parameter j
*x*_**i**_	Variable i
*y*_exp i_(*t*_**n**_)	Experimental value of the *x*_**i**_ variable at time t_n_
*y*_*model*i**_(*t*_**n**_)	Value predicted by the model for *x*_**i**_ variable at time t_n_
$\sigma_{jj}^{2}$	Variance of parameter j
*A*	Physical Activity (counts/min)
*CI*	Confidence Interval
*CVP*	Central Venous Pressure
*DBP*	Diastolic Blood Pressure (mmHg)
δ_**j**_	Sensitive summary
*FIM*	Fisher information matrix
*HR*	Heart Rate (bpm)
*NE*	Norepinephrine (pg/ml)
*Q*	Covariance matrix
*SBP*	Systolic Blood Pressure (mmHg)
*SV*	Stroke Volume (L)
*SVR*	Systemic Vascular Resistance (dyn * s/cm^5^)
*glc*	Glycemia (mmol/L)
*n*	Hill coefficient
