# Supplementary material for: Circadian Rhythm of Blood Pressure of Dipper and Non-dipper Patients With Essential Hypertension: A Mathematical Modeling Approach
Source: Front Physiol. 2021 Jan 18;11:536146. doi: 10.3389/fphys.2020.536146 (PMC7848196; doi:10.3389/fphys.2020.536146)
Supplement: Supplementary file 1 [file Data_Sheet_1.PDF]

Supplementary Material

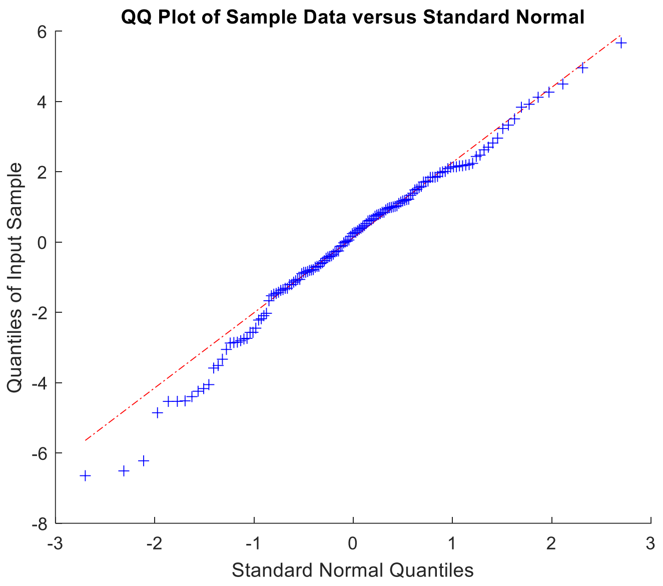

Figure S1. QQ plot of residuals. Residuals were calculated using experimental data and predicted values of the mathematical model.

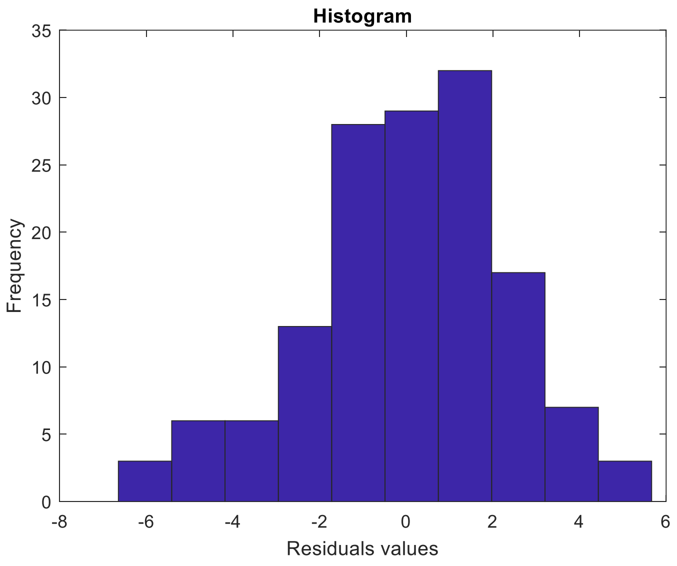

Figure S2. Histogram of residuals. Residuals were calculated using experimental data and predicted values of the mathematical model.
